# An Inaugural Dissertation on Certain Endemic Diseases of Rio de Janeiro: viz. Erysipelas and Hydrocele

**Published:** 1816-06

**Authors:** Joachim Bernardin Pareira, Thomas Seeds


### *An Inaugural Dissertation on certain Endemic Diseases of Rio de Janeiro: viz. Erysipelas and Hydrocele.*
### By Joachim Bernardin Pareira.

( Translated and condensed from the Latin by Thomas Seeds, M. D. )

THE Author of this Dissertation informs us, that the diseases above mentioned, though at present very prevalent, were, seventy years since, almost unknown; and that very old men assert that they themselves had never been attacked by them, nor were they, in their youth, aware that such endemics existed.

How far traditionary information alone is worthy of credit in such a case, we shall not determine. No accurate medical information is to be obtained on the origin of these diseases.

Although the first appearance of these endemics is by no means accounted for, their subsequent prevalence and rapid spread, are attributed to the luxurious and debauched life of the inhabitants; deficient ventilation, want of personal and domestic cleanliness, improper diet, indolence, &c. &c.

Dr. Pareira justly observes, that although man possesses, above all other animals, the power of adaptation to diversity of climate, yet this diversity should never, by the medical philosopher, be overlooked; as it frequently stamps the character, both of the bodily and mental faculties, and likewise determines the nature of the diseases of different regions.

### Medical Topography of Rio de Janeiro.*

Rio de Janeiro is situated in the midst of an extensive plain, nearly surrounded by lofty mountains, and to the westward of a large bay. The city is five miles in extent, and the chain of mountains runs from east to west. Many smaller hills and luxuriant vallies are interspersed amongst the mountains.

The plain on which the city is built, is almost on every side washed by the sea, the shores of which, with little exception, are filthy and disgusting, from receiving the contents of the city sewers. The streets are, for the most part, sufficiently level and very long, but by no means of a commodious or salubrious breadth. The areas and gardens were formerly the receptacles of filth and stagnant water,

---

* Lat. S. 21 deg. 44 min. Long. W. 22 deg. 38 min; and although new roads have since been made, the houses (particularly of the poor) are still very damp. The population of the city is rated at 180,000, and the irregularity of the recently built houses causes the new streets to be as filthy and unhealthy as the old ones. The extreme summer heat is 90° of Fahr. but this intense heat is only perceived in extraordinary seasons, and between the hours of one and three, P. M. The medium summer heat does not usually exceed 74° ; while in the winter, a less degree of heat than 55 is seldom if ever observed.

From this statement it is obvious, that in Rio de Janeiro they have but two seasons, summer and spring. The spring lasts from March till September, while the summer embraces the rest of the year. The atmosphere of the city is generally dry; as, except during the continuance of the violent summer rains, neither oxidation of iron, nor mustiness of vegetables takes place on exposure to the air.

Two periodical winds have been constantly observed at Rio de Janeiro, one of which coming from the northward begins to blow at eleven, P. M.; while the other, an easterly sea breeze, takes the field when its antagonist is at rest: in the summer, it very frequently shifts from east to west, and is then much more powerful than in the spring. Besides these regular and uniform breezes, violent gusts of wind, blowing from different quarters, very variable, and frequently accompanied by thunder storms and torrents of rain, are common occurrences in the summer. These storms are generally preceded by excessive heat, [90°] which they moderate by 10 or 12°, diffusing every where a refreshing coolness. Hail, as might have been expected, is a rare phænomenon. Fogs and mists often occur in the spring, as does small nocturnal rain during the first nights of this season.

Dr. P. conjectures that the vapours, before spoken of, frequently contain noxious exhalations, derived from the marshes and other receptacles of filth and stagnant water. The dews in spring are very copious, but supposed by Dr. Pareira to be of themselves innoxious. The quantity of marsh exhalations is great in proportion to the heat and dryness of the weather; hence the torrents of rain and accompanying high winds, so common in the summer, are believed to be very salutary, as they agitate and purify the air, diffuse an agreeable coolness, promote vegetation, and, by the impulse given to the waves, cause them to take up a vast quantity of the accumulated sordes on the beach, which is so fruitful a cause of evil.

Rio de Janeiro is supplied with water from two sources; one of these is the mountain springs, from whence stone aqueducts are conducted into the city; besides this, river water is brought into the town by wooden pipes, which have only lately (i. e. within these seven years) been laid down. Both the river and spring waters are said to be free from noxious impregnations, and to be perfectly wholesome.

Thus the climate of Rio de Janeiro is mild, serene, and uniform, and the waters good. These circumstances the Doctor asserts, tend to produce a great degree of sensibility and mobility of the nervous system; yet still the habits, moral and physical, of the people are very pernicious. This and many other reasons may reconcile us Hyperboreans to our own uncertain and less genial climate; for without partiality we may maintain, that there is more energy and decision of character, more manly virtue and true happiness among the inhabitants of northern than of southern regions; or, to speak more correctly, amongst the natives of temperate or even cold climates, than of warm ones.

The intense summer heat, the violent changes in the state of the atmosphere, and various moral and physical causes, conspire to produce a tendency to the bilious or sanguineo-bilious temperament; or, to adopt plainer and more accurate language, "the liver is, in Rio de Janeiro, more prone than any other organ to disorder;" a circumstance by no means peculiar to this city, but which it has, in common with many, if not all, tropical towns. Not only the temperament or constitution of the body may be greatly modified, or even altered by the mode of life, but the influence of climate may be materially checked by regimen.

We perceive, that with regard to diet, clothing, lodging, &c. great difference exists amongst the inhabitants of the different regions of Europe; how absurd then, in a climate like that of Rio de Janeiro, to employ the same sort of diet as that used by the inhabitants of the Frigid Zone! Yet such Dr. P. asserts to be the practice of his fellow citizens. "In Rio de Janeiro fere solum alimentum populi regnum animale facit, satis manifesta videbitur ejus diætæ improprietas si natura cœli, temperamentum populi, ejus domesticæ occupationes consideratæ fuerint." Fish too forms a large share of the food of the people. On this subject a long flowery quotation from Cabanis is introduced, which attributes an infinity of evils of every shape and hue to feeding on the inhabitants of the deep. The motto of this gentleman (Cabanis) seems to be, "*Nil mihi cum pelago.*"

It is probably true, as Dr. P. asserts, that his countrymen indulge too much in the use of animal food; he, however, justly concludes, that an unmixed vegetable diet is by no means conducive either to health or strength. In this opinion we decidedly coincide with him, whatever Dr. Lambe and his herbivorous and graminivorous disciples may think of it.*

Spirituous liquors are the ordinary drink of the citizens. Tea and coffee, with which nutmegs, canella alba, and other aromatics have been infused, are drunk very warm and in large quantities. Not only are aromatics employed in this form, but condiments of various kinds are profusely mixed with every sort of solid food.

It is difficult to determine how far the use of aromatics should be carried; for the relaxation and visceral weakness, so universal in warm climates, seem imperiously to demand at least a moderate indulgence in them.†

A practice prevails in Rio de Janeiro, which ought certainly to be universally exploded, we mean that of burying the dead within churches. —The inhabitants of this vicious capital lead very sedentary lives, rarely indulging in innocent recreations in the open air; and are very much addicted to gaming and venereal enjoyments.

In a climate like that of the Brazils, bodily exercise cannot certainly be pushed to the extent it may with us, nor is it necessary it should; but, at the same time, it must be admitted, that there is no stimulus so universal, so efficacious, and so manageable, as exercise; and if the internal organs are overacted on by spices, animal food, and spirituous liquors, while the muscular system is little used, disorders of the circulation and irregularity of the functions must ensue.

The women in easy circumstances use little or no exercise, but that of gestation, excepting at their dances, in which they indulge to excess, drinking at the same time a

---

* Although we do not conceive it necessary *in health* to live entirely on vegetable food, we believe that many diseases might be prevented and cured by this same vegetable diet and aqueous regimen, of which Dr. Seeds entertains so mean an opinion. EDITORS.

† They are as necessary in the *ulterior* stages of an European's residence between the tropics, as they are hurtful before the period of assimilation to the climate is completed. EDITORS.

profusion of warm liquors. On their return from their assemblies, as might be expected, they suffer greatly from sudden exposure to the damp night air. Women of the middle and inferior ranks of society, by indulging in evening walks, suffer a good deal. The people, in general, sit at their doors at night, and sleep with their windows and doors open; of course, not with impunity. Warm bathing, in various forms, is much employed, and is said to do a great deal of harm.* Exposure to moisture the Doctor includes amongst the causes which induce Erysipelas.

It is justly observed, that the state of the cuticular functions depends greatly on the vigour and regularity of the circulation;† and this may be affected by indolence, improper diet, exposure to damp moist air, &c. than which last power, nothing more completely deranges the whole animal machine.

The legs and testicles are parts most frequently attacked by erysipelas; next to these the arms, and lastly the face, which is rarely affected. After a first attack, chronic erysipelas frequently succeeds.

### HYDROCELE.

The prevalence of this disease, Dr. P. attributes to the same causes which give rise to erysipelas; to which may be added, certain other causes acting more locally; viz. immoderate venery, and a certain odious and solitary vice. —The Dissertation is closed with a number of corollaries, which are here subjoined.

I. Men who lead a busy active life, as soldiers, sailors,

---

* That the practice of warm bathing may be carried to effeminacy, and an insalutary excess, amongst a voluptuous, degenerate, and vicious people, cannot be doubted; but that when employed in moderation, it is highly conducive to the regular functions of several important organs in the human frame, both in tropical and hyperborean regions, is equally certain. No organ is more stimulated in hot climates than the skin, and its function is the first to become irregular. If in excess during the day, it is so much the more readily checked by the cold damp air of the evening; in consequence of which, various other functions become deranged. Hence the utility of the evening warm bath in tropical climates, as it draws the circulation to the surface, restores an equable perspiration, and relieves the internal viscera from the oppression under which they labour when the balance of circulation is broken. EDITORS.

† The converse of this is equally true, and much more to be borne in mind for practical purposes; namely, that "The vigour and regularity of the circulation" depends greatly on the state of the cuticular functions. EDITORS.

and many others whose business requires a good deal of walking, are, for the most part, free from both erysipelas and hydrocele.

II. Those who inhabit lofty, airy houses, and are at the same time attentive to daily exercise, enjoy immunity from both these diseases.

III. Those who live in the suburbs, and in houses which are low, but at the same time open and not exposed to marsh effluvia, are perfectly free from these diseases.

IV. Those who use the cold bath only, and abstain from spirituous potations, never suffer.

V. Those who live regularly and frugally, more on vegetable than animal food, likewise escape.

VI. Foreigners (whose mode of life is in general laborious and altogether different from that of the natives) and the blacks are not affected.

VII. Women suffer from erysipelas in Rio de Janeiro much less than men; and this difference is attributed by Dr. Pareira to their abstinence from ardent and spirituous liquors, and their more moderate indulgence in pleasure; the men of this city being, almost from their infancy, devoted to excessive and unnatural indulgences.

Dr. Pareira, the author of this Thesis, was the son of a respectable Portuguese nobleman, and one of those sent to Europe by the Prince Regent of Portugal for the sake of a medical education. He lately paid the debt of Nature at Paris. His manners were mild and engaging; his talents respected; and his habits industrious. He died of Phthisis Pulmonalis.

THOMAS SEEDS, M. D.

